# Metabolomic Insights into the Adaptations and Biotechnological Potential of *Euglena gracilis* Under Different Trophic Conditions

**DOI:** 10.3390/plants14111580

**Published:** 2025-05-22

**Authors:** Sahutchai Inwongwan, Sutthiphat Sriwari, Chayakorn Pumas

**Affiliations:** Department of Biology, Faculty of Science, Chiang Mai University, Chiang Mai 50200, Thailand; sutthiphat_sriwari@cmu.ac.th (S.S.); chayakorn.pumas@gmail.com (C.P.)

**Keywords:** *Euglena gracilis*, metabolomics, trophic modes, ethanol metabolism, bioactive compounds

## Abstract

*Euglena gracilis* is a metabolically versatile microalga capable of thriving under photoautotrophic (light, no ethanol), mixotrophic (light with 1% *v*/*v* ethanol), and heterotrophic (dark with 1% *v*/*v* ethanol) conditions. Here, we applied untargeted LC-MS metabolomics (Agilent 1290 LC, 6545XT QTOF-MS; Agilent Technologies, Santa Clara, California, USA) to investigate its trophic-mode-dependent metabolic adaptations and assess its biotechnological potential. Metabolites were separated on a C18 column and analyzed in both positive and negative ion modes. Multivariate analyses (PCA and sPLS-DA) revealed clear and reproducible metabolic separations among growth modes (*p* < 0.001). Photoautotrophic cultures were enriched in phenolic acids, flavonoids, and lipid classes associated with oxidative stress protection. Mixotrophy induced a broader spectrum of upregulated metabolite classes, including saccharolipids, macrolactams, and triterpenoids, reflecting a hybrid metabolism combining photosynthesis and ethanol utilization. Heterotrophic cultures showed elevated levels of polyamines and amino acids (e.g., putrescine, proline), indicative of redox regulation and stress adaptation in dark, ethanol-rich conditions. Class-level comparisons identified distinct and shared metabolite categories, with photoautotrophy favoring antioxidant biosynthesis and mixotrophy supporting metabolic diversity. These findings provide metabolite-level insights into the extraordinary plasticity of *E. gracilis* and offer a framework for optimizing cultivation strategies to enhance the targeted production of high-value bioproducts.

## 1. Introduction

Microalgae exhibit remarkable adaptability to varying environmental conditions through phenotypic plasticity, genetic variation, and symbiotic interactions, making them valuable models for understanding stress tolerance and informing sustainable practices in aquaculture, ecosystem management, and environmental monitoring. Among them, *Euglena gracilis* stands out for its exceptional metabolic plasticity, growing under autotrophic, mixotrophic, and heterotrophic conditions [1,2,3]. This adaptability is driven by its ability to utilize diverse carbon sources, such as ethanol, glucose, glutamate, succinate, and pyruvate [4,5]. Additionally, its resilience across a wide pH range and tolerance to various environmental stressors demonstrate its robustness and industrial potential. These features make *E. gracilis* a valuable candidate for applications in biofuels, functional foods, pharmaceuticals, and environmental biotechnology.

The metabolic versatility of *E. gracilis* stems from its ability to thrive under diverse trophic conditions and utilize a wide range of organic substrates. This complexity is reflected in its large, highly intricate genome, which poses challenges for comprehensive molecular annotation. Transcriptomic studies have revealed biosynthetic pathways for carotenoids, isoprenoids, fatty acids, and thylakoid glycolipids, as well as genes involved in stress response and signalling [6]. Wax ester fermentation, a key feature of anaerobic metabolism, appears to be regulated post-transcriptionally, indicating additional layers of control expression [7]. Mitochondrial genome analysis confirms its capacity for both aerobic and anaerobic respiration [8], while subcellular enzyme localization underscores the compartmentalization of its metabolism [9]. Notably, its plastid proteome lacks oxidative pentose phosphate pathway enzymes, distinguishing it from typical chloroplast-containing algae and suggesting unique redox regulation [10].

A growing body of research highlights *E. gracilis* as a promising biomanufacturing platform due to its ability to produce high-value bioactive metabolites, including carotenoids, wax esters, phenolic compounds, shikimic acid, and vitamins A, C, and E [11]. These metabolites exhibit antioxidant, antimicrobial, and anti-inflammatory properties, positioning *E. gracilis* as a key resource for nutraceutical and pharmaceutical applications [12]. Additionally, *E. gracilis* harbors a vast, yet largely untapped repertoire of secondary metabolites with potential pharmacological significance. Recent metabolomic studies have revealed novel terpenoids, alkaloids, and phenolic glycosides with bioactive properties, including anti-inflammatory, neuroprotective, and antimicrobial effects [13]. Compounds such as euglenophycin, a cytotoxin with antimicrobial potential, and sulfolipids, exhibiting antiviral activity, highlight the biochemical diversity within *E. gracilis* [14].

Recent metabolomics studies have revealed how *E. gracilis* reprograms its metabolism under different trophic conditions. Untargeted NMR and high-resolution MS approaches have shown that heterotrophic cells accumulate higher levels of polar amino acids and flavonoid-like polyphenolics, while photoheterotrophic cultures are enriched in carbohydrates, alkaloids, and organoheterocyclic metabolites [15,16]. Photoautotrophic growth supports the accumulation of monosaccharides, storage lipids, and specific alkaloids, whereas mixotrophy enhances the synthesis of amino acids, peptides, and long-chain polyunsaturated fatty acids [17]. Under nitrogen deprivation, targeted metabolomics has revealed increased fatty acid content with a concurrent reduction in chlorophyll and protein levels [18]. Anaerobic conditions further redirect carbon from paramylon toward wax ester biosynthesis and reconfigure sulfur metabolism, leading to glutathione degradation and accumulation of sulfur-containing amino acids and H_2_S [19]. Collectively, these findings underscore the organism’s capacity to modulate lipid, carotenoid, and secondary metabolite pathways in response to trophic and environmental changes. To fully capture this metabolic flexibility, integrated omics approaches combining metabolomics with transcriptomics and proteomics are increasingly employed [20].

Trophic mode strongly influences metabolic pathways and bioactive compound production in microalgae. Autotrophic growth supports photosynthesis-driven metabolism but is constrained by light availability, whereas heterotrophy enables higher biomass yields by shifting energy metabolism toward the oxidation of external organic carbon sources [21,22]. *E. gracilis* is uniquely capable of mixotrophy, combining CO_2_ fixation with organic substrate utilization to enhance both growth and metabolic diversity [6,23,24]. Understanding how these trophic strategies shape metabolite profiles is key to harnessing *E. gracilis* for industrial applications.

Trophic mode plays a critical role in shaping the metabolic landscape of *E. gracilis*. While organic carbon sources like ethanol can influence central metabolism by enhancing NADH supply and TCA cycle activity [9,25], their broader impact under different trophic conditions remains underexplored. This study employs untargeted metabolomics to systematically investigate metabolic adaptations of *E. gracilis* under autotrophic, mixotrophic, and heterotrophic growth. By identifying condition-specific metabolic shifts and bioactive compound production, we offer new insights into how trophic modes drive functional versatility in *E. gracilis*, supporting its potential in functional food development, health-related applications, and sustainable bioproducts. These findings also contribute to broader efforts toward sustainable resource utilization and bioinnovation, in line with global goals for improving food security, health, and clean energy development.

## 2. Results

### 2.1. Flavonoid and Phenolic Content Across Trophic Conditions

**Figure 1:**
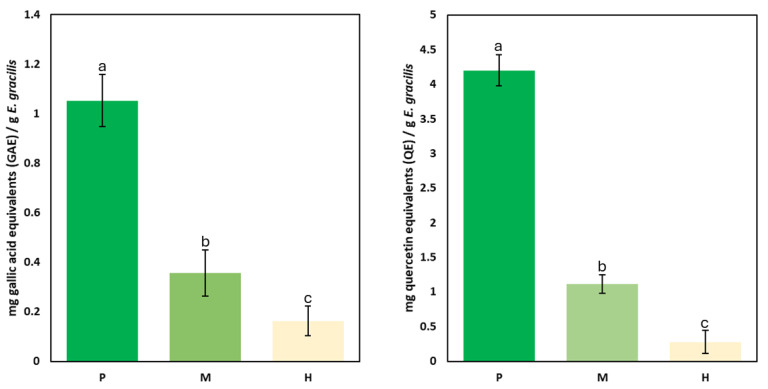
Total phenolic (**left**) and flavonoid (**right**) content in *E. gracilis* cultivated under photoautotrophic (P), mixotrophic (M), and heterotrophic (H) conditions. Phenolic content is expressed as mg gallic acid equivalents (GAE) per g dry weight, and flavonoid content as mg quercetin equivalents (QE) per g dry weight. Statistical analysis was performed using one-way ANOVA followed by Tukey’s HSD post hoc test. Different letters (a, b, c) indicate significant differences between groups at *p* < 0.05.

*E. gracilis* cultures grown for 10 days under photoautotrophic, mixotrophic, and heterotrophic conditions exhibited distinct growth profiles, reaching approximately 0.7, 1.2, and 1.8 million cells/mL, respectively. These values correspond to the late exponential phase, suitable for metabolic analysis. Total phenolic and flavonoid contents were then quantified in cultures grown under each condition (Figure 1). The results indicate that autotrophic conditions significantly enhance the accumulation of both phenolic and flavonoid compounds, while heterotrophic growth leads to the lowest concentrations. In the phenolic content analysis (left panel), autotrophic cultures exhibited the highest phenolic accumulation, reaching approximately 1.05 mg/g, followed by mixotrophic cultures at around 0.34 mg/g, while heterotrophic cultures contained the lowest amount at approximately 0.16 mg/g. This trend suggests that photosynthetic activity plays a crucial role in phenolic biosynthesis, possibly due to the involvement of phenylpropanoid pathways that are stimulated by light exposure and oxidative stress protection mechanisms [26].

A similar trend was observed in the flavonoid content (right panel), where autotrophic conditions resulted in the highest flavonoid accumulation (4.20 mg/g), followed by mixotrophic cultures (1.11 mg/g), and heterotrophic conditions (0.28 mg/g). The significantly lower flavonoid content under heterotrophy suggests that flavonoid biosynthesis is highly dependent on light-driven metabolic pathways. These results highlight light availability as a major driver of phenolic and flavonoid production in *E. gracilis*, with autotrophic conditions promoting higher accumulation. This suggests that light-regulated pathways play a central role in secondary metabolism [27]. To better understand these mechanisms, the following section explores the underlying metabolic pathways influenced by trophic mode.

### 2.2. Metabolomics Overview and Global Metabolic Variability

**Figure 2:**
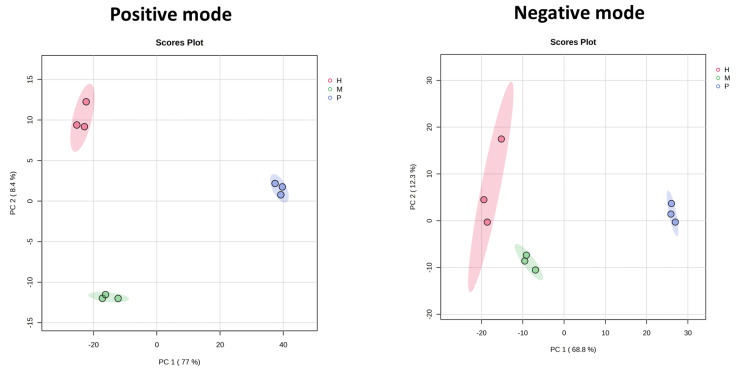
Principal Component Analysis (PCA) of metabolic profiles of *E. gracilis* under different trophic conditions. Left: positive ionization mode; right: negative ionization mode. Each dot represents an individual biological replicate (n = 3 per condition), and ellipses indicate 95% confidence intervals for each group. Photoautotrophic (P), mixotrophic (M), and heterotrophic (H) samples show clear separation along PC1 and PC2, reflecting distinct metabolic profiles associated with each trophic mode.

Principal Component Analysis (PCA) was conducted to explore global metabolic variation in *E. gracilis* under photoautotrophic, mixotrophic, and heterotrophic conditions. Figure 2 presents unsupervised PCA score plots of *E. gracilis* metabolic profiles under photoautotrophic (P), mixotrophic (M), and heterotrophic (H) conditions, analyzed in positive (left) and negative (right) ionization modes. In the positive mode, PC1 and PC2 explain 77.0% and 8.4% of the total variance, respectively, while in the negative mode, they account for 68.8% and 12.3%. Clear separation among the three trophic conditions along PC1 indicates that trophic mode is the dominant source of metabolic variation. PC2 further resolves sample clusters within each group, supporting the reproducibility of biological replicates and highlighting distinct metabolic states under each growth condition. In both ionization modes, heterotrophic samples are well separated from autotrophic samples, while mixotrophic samples occupy an intermediate position, suggesting partial overlap with both groups. Differences in the variance and clustering patterns between positive and negative modes reflect ionization-specific metabolite contributions but consistently support trophic-mode-dependent metabolic divergence. PCA thus effectively demonstrates that metabolic composition in *E. gracilis* is strongly shaped by trophic condition.

**Figure 3:**
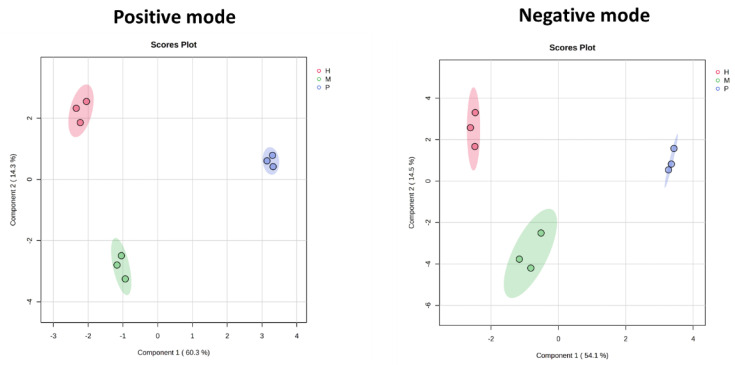
Sparse Partial Least Squares Discriminant Analysis (sPLS-DA) score plots of *E. gracilis* metabolic profiles under different trophic conditions. Left: positive ionization mode; right: negative ionization mode. sPLS-DA reveals clear group separation among photoautotrophic (P), mixotrophic (M), and heterotrophic (H) conditions, indicating distinct sets of discriminant metabolites associated with each trophic mode.

To enhance group separation and identify key metabolites driving the observed differences, Sparse Partial Least Squares Discriminant Analysis (sPLS-DA) was performed following PCA. This supervised method enables clearer discrimination between trophic conditions and highlights metabolites most associated with each metabolic profile (*p* < 0.001). Figure 3 shows sPLS-DA score plots for positive (left) and negative (right) ionization modes, with Component 1 explaining 60.3% and 54.1% of the variance, respectively. Compared to PCA, sPLS-DA provided improved separation among photoautotrophic, mixotrophic, and heterotrophic conditions, confirming distinct metabolic profiles associated with each trophic mode. In both modes, heterotrophic samples clustered the farthest from autotrophic samples, indicating the most substantial metabolic divergence. Mixotrophic samples remained intermediate, reflecting a blended metabolic state. The stronger separation along Component 1 in positive mode suggests that metabolites captured in this ionization mode play a larger role in differentiating trophic conditions, while Component 2 in negative mode contributed additional discriminatory power. Together, these results support clear trophic mode-specific metabolic signatures in *E. gracilis*.

### 2.3. Heatmap and Clustering Analysis of Metabolic Profiles

**Figure 4:**
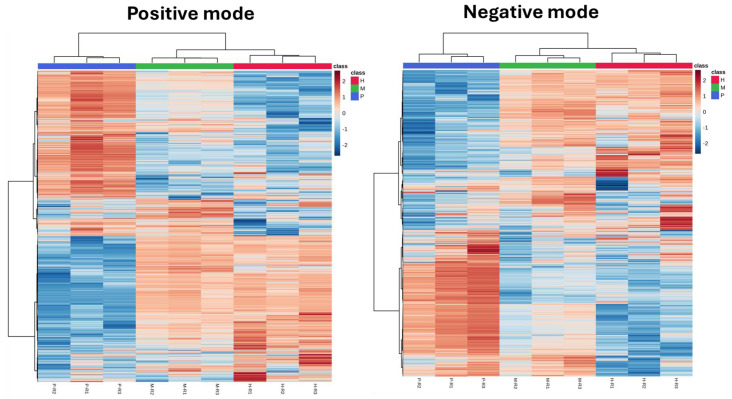
Heatmap analysis of metabolite abundance in *E. gracilis* under different trophic conditions. Left: positive ionization mode; right: negative ionization mode. Each column represents an individual replicate labeled as P-R1 to P-R3 (photoautotrophic), M-R1 to M-R3 (mixotrophic), and H-R1 to H-R3 (heterotrophic). Red indicates higher abundance, while blue denotes lower abundance. Hierarchical clustering reveals distinct, condition-specific metabolic patterns, confirming reproducible shifts across biological replicates.

To examine the global metabolite distribution across different trophic modes in *E. gracilis*, hierarchical clustering heatmaps were generated for both positive and negative ionization mode datasets (Figure 4). Each heatmap represents standardized metabolite abundance, with red indicating higher intensity and blue indicating lower intensity. The analysis revealed clear and reproducible clustering patterns that reflect distinct metabolic states under photoautotrophic, mixotrophic, and heterotrophic conditions. In both ionization modes, photoautotrophic samples formed a separate and coherent cluster, indicating a distinct metabolic configuration under light-dependent growth. The consistency of this separation across both ion modes suggests that photoautotrophy induces a unique metabolic program, distinguishable at the global scale. In contrast, mixotrophic and heterotrophic samples showed greater similarity in their clustering profiles, with closer proximity and overlapping patterns in metabolite abundance. This trend was more pronounced in the positive ionization mode, where M and H samples exhibited shared intensity gradients across multiple metabolite groups. While differences were evident across all three trophic states, the heatmap gradients suggest that mixotrophic samples possess a blended metabolic signature, showing partial overlap with both P and H groups but leaning more closely toward H. This proximity implies a stronger influence of organic carbon utilization—likely due to ethanol or other substrate uptake—on the mixotrophic metabolic profile.

**Figure 5:**
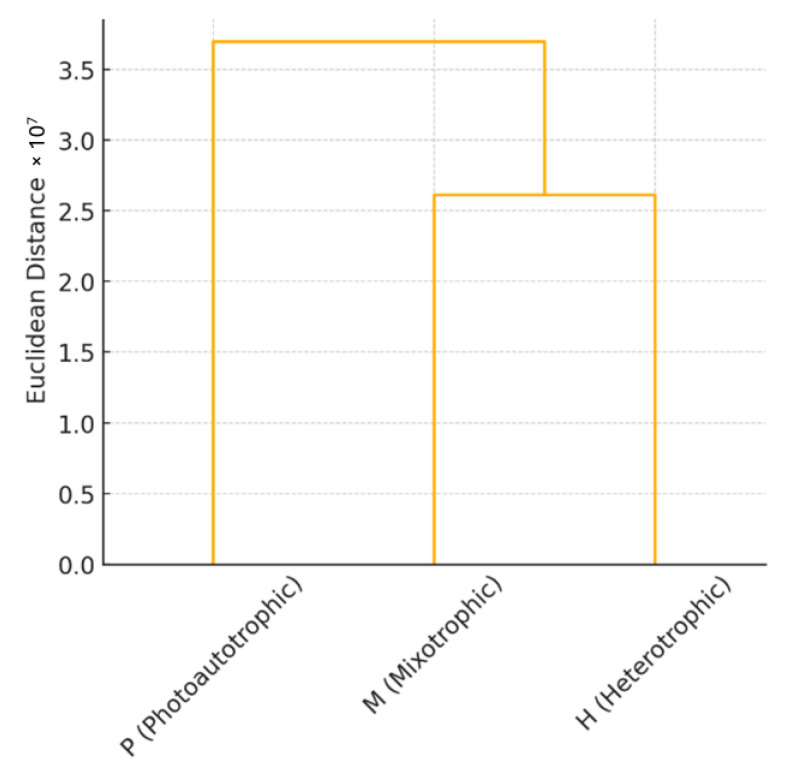
Neighbor-joining clustering of *E. gracilis* metabolic profiles under different trophic conditions based on Euclidean distance. Hierarchical clustering was performed using combined positive and negative mode datasets to assess metabolic similarity across photoautotrophic (P), mixotrophic (M), and heterotrophic (H) conditions. Euclidean distance values indicate the magnitude of metabolic divergence.

To further assess the global metabolic similarity among trophic modes, a neighbor-joining dendrogram was constructed based on Euclidean distances using the combined metabolomic datasets from both ionization modes (Figure 5). The dendrogram quantitatively visualizes the degree of dissimilarity between photoautotrophic (P), mixotrophic (M), and heterotrophic (H) conditions. Among the three, photoautotrophic samples were metabolically the most distinct, with the largest Euclidean distance (~3.7 × 10^7^) from heterotrophic samples. Mixotrophic samples clustered more closely with heterotrophic samples (distance ~2.6 × 10^7^) than with photoautotrophic samples (distance ~3.6 × 10^7^), reinforcing previous findings that mixotrophic metabolism shares greater similarity with heterotrophic than autotrophic states. This pattern supports the notion that in the presence of ethanol, mixotrophic cells shift their metabolic configuration toward a more heterotrophic-like profile despite being cultured in light. Overall, the clustering pattern aligns with prior heatmap and multivariate analyses, providing consistent evidence for trophic-mode-dependent metabolic divergence in *E. gracilis*. These results further support the idea that mixotrophy represents an intermediate but asymmetrically aligned state, metabolically closer to heterotrophy.

### 2.4. Venn Diagram Analysis Reveals Trophic-Specific Metabolites in E. gracilis

To ensure a high-confidence metabolomics dataset, stringent filtering criteria were applied, including a signal-to-noise (S/N) ratio ≥ 10, coefficient of variation (CV%) ≤ 30%, and response intensity ≥ 10,000. These parameters eliminated low-abundance and high-variance features, improving the reliability of detected metabolites. After filtering, 1129 and 1433 metabolite peaks were identified in positive and negative ionization modes, respectively, with only 75 overlapping metabolites. This limited overlap reflects the complementary strengths of dual-mode acquisition in capturing a broader metabolome. The complete list is provided in Supplementary Data S1. The greater number of metabolites in negative mode suggests its enhanced sensitivity toward polar and acidic compounds, while positive mode favors nonpolar, lipophilic metabolites. Pathway analysis was subsequently performed on the combined dataset to investigate condition-specific metabolic patterns.

**Figure 6:**
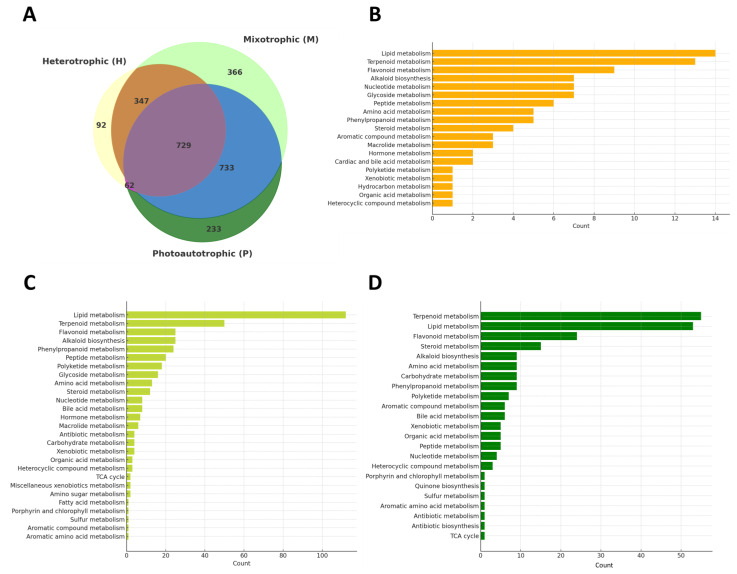
Comparative metabolomic analysis of *E. gracilis* under different trophic modes. The Venn diagram showing the number of shared and unique metabolites detected in *E. gracilis* under photoautotrophic, mixotrophic, and heterotrophic conditions based on combined positive and negative ionization mode datasets (**A**), alongside pathway enrichment analysis of metabolites uniquely identified under heterotrophic (**B**), mixotrophic (**C**), and photoautotrophic (**D**) conditions, with bar plots indicating the number of metabolites assigned to each metabolic pathway category.

A three-way Venn diagram (Figure 6A) compared metabolite profiles under photoautotrophic, mixotrophic, and heterotrophic conditions. Of the total detected features, 729 metabolites were shared across all conditions, representing a conserved core metabolic network. Unique metabolite sets were identified for each condition—233 in photoautotrophy, 366 in mixotrophy, and 92 in heterotrophy—reflecting distinct metabolic responses to environmental inputs. To further characterize these condition-specific features, pathway enrichment analysis was performed separately for each mode (Figure 6B–D). The results highlight unique metabolic signatures associated with each growth strategy. Full metabolite lists and pathway assignments are available in Supplementary Data S1. These findings illustrate the metabolic adaptability of *E. gracilis* in response to differing trophic environments.

Under heterotrophic conditions, 92 unique metabolites were enriched predominantly in lipid metabolism, followed by terpenoid, flavonoid, and alkaloid metabolism (Figure 6B). Additional but less prominent enrichment was observed in glycoside, peptide, amino acid, and steroid metabolism. This suggests that in the absence of light, *E. gracilis* prioritizes lipid and secondary metabolite synthesis pathways, potentially to maintain membrane integrity and redox balance via ethanol-derived carbon flux.

In the mixotrophic condition, which yielded the highest number of unique metabolites (366), enrichment was broadly distributed across multiple metabolic categories (Figure 6C). Lipid metabolism remained the dominant pathway, with strong representation also in terpenoid, flavonoid, alkaloid, and glycoside metabolism. This pattern supports a synergistic metabolic state driven by simultaneous access to light and ethanol, enhancing both energy production and biosynthesis of secondary metabolites. Notably, there was extended coverage of peptide, amino acid, nucleotide, and steroid metabolism, indicating a metabolically versatile and responsive profile.

For photoautotrophic conditions, 233 unique metabolites were identified, with a strong bias toward terpenoid metabolism, followed by lipid metabolism, flavonoid metabolism, and contributions from steroid and alkaloid biosynthesis (Figure 6D). The enrichment in porphyrin, amino acid, and carbohydrate metabolism reflects active primary metabolism associated with photosynthetic activity, while xenobiotic-related and antioxidant pathways suggest responses to light-induced oxidative stress.

Across all conditions, terpenoid metabolism remains a consistently enriched and dominant pathway, showing its central role in *E. gracilis* physiology. However, the intensity and context of this enrichment differ by trophic mode, supporting its regulation by light availability and carbon source. Lipid metabolism is also universally active but is most pronounced under mixotrophic conditions, correlating with enhanced metabolite diversity and known patterns of lipid accumulation when both light and ethanol are available. This comparative analysis reveals trophic mode-specific metabolic rewiring. Photoautotrophy predominantly enriches pathways tied to photosynthetic function and stress response, such as terpenoid, flavonoid, and porphyrin metabolism. In contrast, mixotrophy activates a broader array of biosynthetic routes, including extensive lipid, flavonoid, glycoside, and alkaloid metabolism, reflecting a metabolically flexible state fueled by dual energy inputs. Meanwhile, heterotrophic metabolism is more focused, with enrichment concentrated in lipid, terpenoid, and flavonoid pathways, suggesting adaptation to dark, ethanol-driven conditions that favor membrane and antioxidant biosynthesis. Collectively, these patterns (Figure 6) reinforce the remarkable metabolic plasticity of *E. gracilis*, enabling it to dynamically adjust its biochemical landscape in response to environmental cues and nutrient availability.

### 2.5. Differential Metabolite Analysis

**Figure 7:**
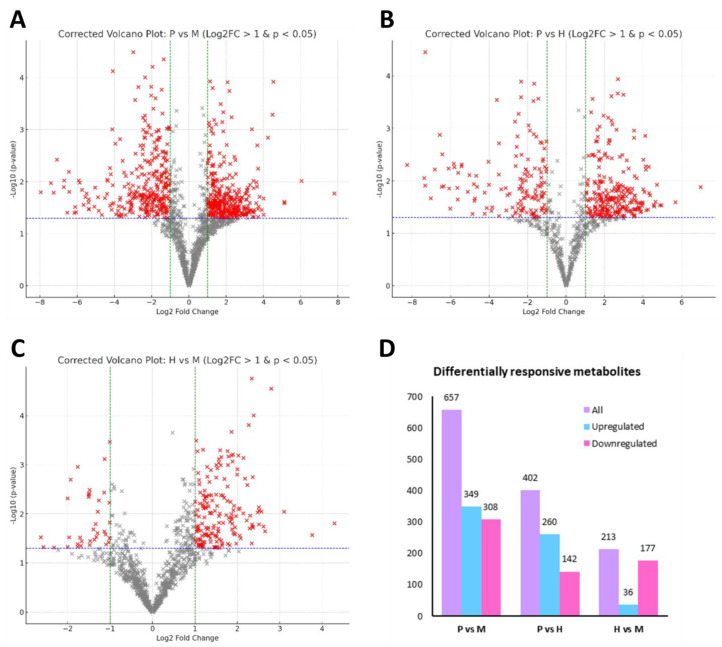
Differential metabolite profiles of *E. gracilis* under photoautotrophic (P), mixotrophic (M), and heterotrophic (H) conditions. Volcano plots illustrate significantly altered metabolites (red crosses; *p* ≤ 0.05, |log_2_FC| > 1) for each pairwise comparison: P vs. M (**A**), P vs. H (**B**), and H vs. M (**C**). Grey crosses indicate non-significant features that do not meet the fold-change or *p*-value thresholds. Panel (**D**) summarizes the number of total, upregulated, and downregulated metabolites identified in each comparison.

To examine metabolic shifts among trophic conditions, volcano plots (Figure 7) were generated for pairwise comparisons between photoautotrophic, mixotrophic, and heterotrophic conditions. Metabolites with a log_2_ fold change (FC) > 1 and adjusted *p*-value (adj *p* ≤ 0.05) were considered significantly upregulated or downregulated. The top 50 differentially expressed metabolites from each comparison, ranked by absolute log_2_ fold change, are provided in Supplementary Data S2.

The comparison between autotrophic and mixotrophic conditions (P vs. M) revealed the highest number of differentially expressed metabolites, with 349 upregulated and 308 downregulated metabolites in mixotrophic conditions (Figure 7A). The upregulated metabolites were associated with lipid metabolism, phenolic biosynthesis, and ethanol-related metabolic pathways, indicating a substantial metabolic shift due to the combined influence of photosynthesis and ethanol supplementation. In the autotrophic vs. heterotrophic comparison, 260 metabolites were upregulated and 142 were downregulated (Figure 7B), reflecting a shift toward terpenoid biosynthesis, flavonoid metabolism, and stress-responsive phenolic compounds. The increase in secondary metabolite production suggests that heterotrophy induces adaptive metabolic pathways in response to ethanol as the sole carbon source. Meanwhile, the mixotrophic vs. heterotrophic comparison displayed a different pattern, with 177 downregulated and only 36 upregulated metabolites under heterotrophic conditions (Figure 7C). The dominance of downregulated metabolites suggests that heterotrophy results in a metabolic simplification compared to mixotrophy where fewer active pathways are maintained, possibly due to the loss of photosynthetic activity.

The bar plot in Figure 7D summarizes the number of upregulated and downregulated metabolites across comparisons. The P vs. M comparison exhibited the most pronounced metabolic alterations, reinforcing that mixotrophy induces the largest metabolic shift compared to autotrophy. The P vs. H comparison showed a similar trend, but with fewer metabolites involved, reflecting the loss of photosynthesis and the reliance on ethanol-driven metabolism under heterotrophic conditions. In contrast, the M vs. H comparison exhibited the fewest upregulated metabolites, suggesting that mixotrophic and heterotrophic metabolism share more similarities than autotrophic and heterotrophic states.

For each pairwise comparison between trophic conditions, the top 20 metabolite classes were selected based on their absolute log_2_ fold change (|log_2_FC|). Classes were ranked according to |log_2_FC| values, and the 20 most differentially regulated classes were extracted to facilitate comparative profiling. This selection approach enabled a consistent focus on the most strongly upregulated or downregulated metabolic categories across conditions. Subsequent analyses compared the identity, overlap, and unique enrichment patterns of these top 20 classes among photoautotrophic, mixotrophic, and heterotrophic samples to elucidate trophic mode-specific metabolic reprogramming.

**Figure 8:**
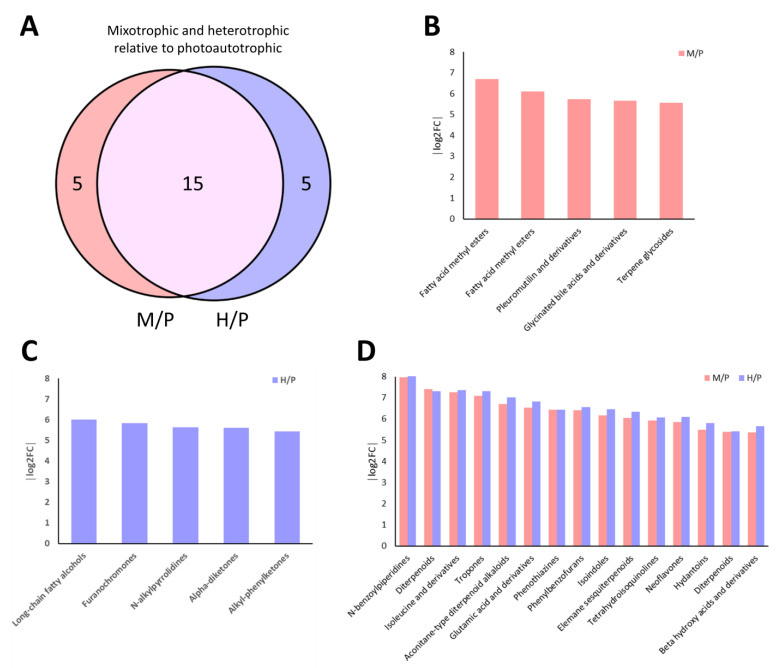
Shared and unique metabolite classes enriched under mixotrophic (M) and heterotrophic (H) conditions relative to photoautotrophy (P). The central Venn diagram (A) shows overlapping and unique metabolite classes from M/P and H/P comparisons, while the accompanying bar plots display the top five upregulated classes unique to mixotrophy (**B**), heterotrophy (**C**), and those commonly enriched across both conditions (**D**).

To further understand the impact of ethanol supplementation under light (mixotrophy) and dark (heterotrophy) conditions, we compared highly enriched metabolite classes relative to photoautotrophic growth (Figure 8). A total of 25 metabolite classes were significantly upregulated in either or both M/P and H/P comparisons, with 15 classes shared between the two, and 5 classes uniquely enriched in each condition (Figure 8A). In mixotrophic conditions (M/P), fatty acid methyl esters, glycinated bile acids, phenolamides, and terpene glycosides were among the top enriched classes (Figure 8B), suggesting enhanced lipid remodeling and antioxidant responses. Meanwhile, heterotrophic conditions (H/P) favored enrichment of long-chain fatty alcohols, furanocoumarins, and nitrogen-containing bioactives such as N-alkylpyrrolidines and alkyl-phenylketones, indicating a shift toward ethanol-derived metabolism and detoxification pathways (Figure 8C). Notably, several shared metabolite (Figure 8D) classes such as diterpenoids, tropanes, and isoalkenes showed consistent upregulation in both M/P and H/P comparisons, highlighting common ethanol-responsive metabolic pathways.

**Figure 9:**
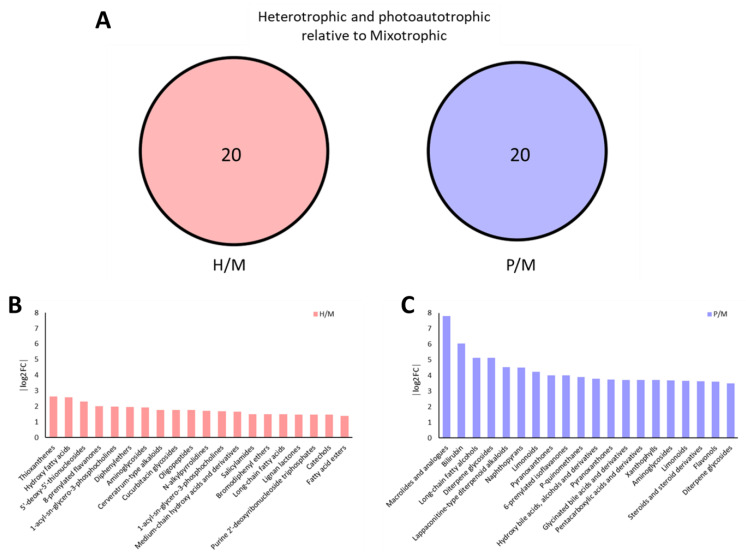
Metabolite classes uniquely enriched in heterotrophic (H) and photoautotrophic (P) conditions relative to mixotrophy (M). The Venn diagram (**A**) illustrates the overlap and unique metabolite classes from H/M and P/M comparisons, while the bar plots highlight the top classes specifically enriched in heterotrophic (**B**) and photoautotrophic (**C**) conditions.

The Venn diagram in Figure 9A shows that 20 metabolite classes were unique to P/M (P vs. M), while 20 were unique to H/M (H vs. M), with no overlap, suggesting distinct metabolic rewiring in response to ethanol-driven heterotrophy or combined photo–ethanol metabolism. In the P/M comparison, autotrophy significantly upregulated macrolides and analogs, bilirubin, long-chain fatty alcohols, and lappaconitine-type alkaloids (log_2_FC > 5) (Figure 9B), indicating enhanced oxidative stress protection, membrane stabilization, and terpenoid biosynthesis under light conditions. Notably, various oxygenated lipids and flavonoid derivatives, including aminophenols, anthoxanthins, and xanthophylls, were also higher in autotrophy, underscoring the antioxidant-rich profile of light-grown cells. In contrast, the H/M comparison revealed that heterotrophic cultures upregulated thioxanthenes, 5′-deoxythionucleosides, and hydroxy fatty acids (log_2_FC ~2.0), along with several classes involved in nucleoside and lipid metabolism, such as salicylamides and bromodiphenyl ethers (Figure 9C). The relatively lower log_2_FCs in the H/M comparison reflect a more constrained metabolic profile under full heterotrophy compared to mixotrophy, where energy and metabolite fluxes are more diverse due to simultaneous use of light and ethanol.

**Figure 10:**
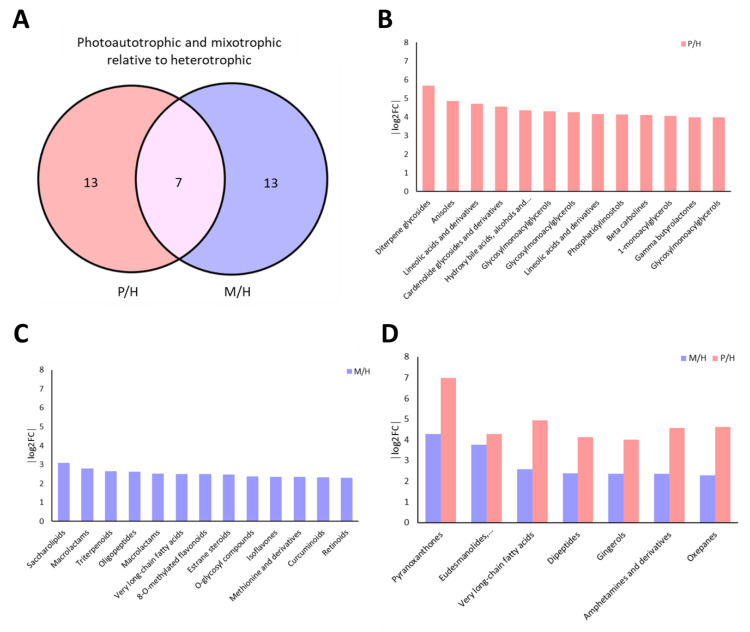
Distinct and shared metabolite classes in photoautotrophic (P) and mixotrophic (M) conditions relative to heterotrophy (H). The Venn diagram (**A**) illustrates unique and overlapping upregulated metabolite classes from P/H and M/H comparisons. Bar plots display the top enriched classes in photoautotrophic (**B**) and mixotrophic (**C**) conditions, while panel (**D**) highlights shared classes upregulated in both comparisons.

Comparative analysis of metabolite classes between photoautotrophic (P), mixotrophic (M), and heterotrophic (H) conditions revealed 33 upregulated classes in total—13 unique to P/H, 13 to M/H, and 7 shared (Figure 10A). In P/H, the most enriched classes included diterpene glycosides, alkaloids, and linoleic acid derivatives, all associated with antioxidant or photoprotective functions (Figure 10B). Additional upregulated classes related to lipid remodeling (e.g., glycosylmonoacylglycerols, phosphoethanolamines) suggest active adaptation to light-induced oxidative stress.

In M/H, saccharolipids, macrolactams, and triterpenoids were among the top upregulated classes, indicating activation of both lipid and secondary metabolite pathways under ethanol-supplemented, light-grown conditions (Figure 10C). Although fold changes were generally lower than in P/H, mixotrophy supported a broader diversity of enriched classes. Shared classes such as pyranoxanthones and very long-chain fatty acids (Figure 10D) showed higher fold changes in P/H, reinforcing the stronger metabolic shift under photoautotrophy. These results demonstrate distinct regulatory patterns shaped by trophic mode and highlight potential antioxidant-rich metabolites favored under light-based cultivation.

Metabolomic comparisons of *E. gracilis* under photoautotrophic, mixotrophic, and heterotrophic conditions revealed distinct metabolic adaptations relative to each trophic state. When compared to the photoautotrophic condition, both mixotrophic and heterotrophic cells shared 15 commonly upregulated metabolites, including N-benzoylpiperidines, isoflavonoids, phenylthiazoles, and other secondary metabolites, suggesting conserved adaptive responses under ethanol-supplemented systems. Unique to the mixotrophic state were fatty acid methyl esters, glycated bile acids, and terpene glycosides, indicating an intermediate metabolic strategy that incorporates lipid remodeling and ethanol utilization. In contrast, heterotrophic cells uniquely produced long-chain fatty alcohols, furanocoumarins, and N-alkylpyrrolidines, pointing to a deeper shift toward nitrogen metabolism and stress-associated bioactives when photosynthesis is absent.

Relative to mixotrophy, both photoautotrophic and heterotrophic cells expressed distinct metabolite sets. The photoautotrophic condition showed upregulation of macrolides, lappaconitine-type alkaloids, hydroxy bile acids, and xanthophylls, emphasizing its reliance on photosynthesis-associated antioxidants and secondary metabolite production. Conversely, heterotrophic cells overproduced thioxanthenes, aminoglycosides, deoxyinosine triphosphates, and brominated compounds, suggesting a shift toward nucleoside metabolism and ethanol-linked energy pathways. Notably, each condition displayed 20 unique top-ranking metabolites in these pairwise comparisons, reflecting their metabolic divergence.

When heterotrophy was used as the reference, both photoautotrophic and mixotrophic cells shared upregulated metabolites related to antioxidant defense and lipid metabolism, such as saccharolipids, curcuminoids, and flavonoids. Photoautotrophy uniquely expressed diprenyl flavonoids, carotenoids, and glycosylmonoacylglycerols, reinforcing its role in light-driven oxidative stress mitigation. Mixotrophic cells, meanwhile, favored saccharolipids, macrocyclic lactams, and O-glycosyl compounds, reflecting hybrid metabolic activity. Overall, the analysis highlights that mixotrophy integrates features of both autotrophy and heterotrophy, balancing ethanol assimilation with the retention of bioactive metabolites typically associated with photosynthetic function. Heterotrophy distinctly shifts toward nitrogenous and nucleoside metabolites, while autotrophy supports the accumulation of photosynthesis-related antioxidants and secondary metabolites.

## 3. Discussion

### 3.1. Global Metabolic Shifts Under Different Trophic Modes and Metabolic Reprogramming in Response to Ethanol and Light Availability

Untargeted LC-MS analysis revealed distinct metabolic profiles of *E. gracilis* under photoautotrophic, mixotrophic, and heterotrophic conditions. PCA and sPLS-DA showed clear, reproducible separations, indicating strong metabolic reprogramming driven by light availability and ethanol supplementation. These multivariate patterns align with prior observations that *E. gracilis* can significantly rearrange its metabolism in response to different nutritional modes [28]. This is consistent with recent metabolomic studies reporting 137 metabolites differing between autotrophic and mixotrophic *E. gracilis* cultures and similarly distinct metabolomes under light vs. dark growth [20]. Heatmap clustering of metabolite abundance further illustrates global shifts in metabolic states, emphasizing that both light and ethanol act as dominant regulators of metabolic flux.

Some studies have reported key *Euglena* metabolite markers such as zeatin derivatives [29]. In our dataset, targeted ion searches confirmed the presence of melatonin [30] (detected in both positive and negative modes) and jasmonic acid [31] (in negative mode), suggesting activation of hormonal signaling under specific trophic conditions. However, other previously reported metabolites—including zeatins, 5-hydroxytryptamine, and beta-carotene—were not confidently detected above our quality thresholds. Their absence may be due to low abundance under the conditions tested, limited extraction efficiency with 70% methanol, or suboptimal ionization under our ESI-QTOF parameters.

Under photoautotrophic conditions, cells prioritized metabolism for photosynthesis and photoprotection. Consistent with earlier reports, autotrophic *Euglena* accumulates monosaccharides and specific membrane lipids rather than large storage reserves [17]. Nevertheless, autotrophic cells in our study did synthesize baseline levels of storage and secondary metabolites, indicating a balanced metabolism tuned to steady, light-driven growth. Mixotrophic growth (light plus ethanol) produced a distinct metabolic signature suggestive of a hybrid physiology. Interestingly, we observed that mixotrophic *E. gracilis* did not simply sum the features of autotrophic and heterotrophic modes, but rather exhibited unique shifts. Previous studies have reported that mixotrophic *E. gracilis* can display diminished photosynthetic efficiency by the late growth phase [17], presumably because the cells prioritize oxidation of external carbon (ethanol) over light-driven carbon fixation.

The mixotrophic metabolome also featured abundant long-chain fatty acids (including polyunsaturated fatty acids, PUFAs), which were less prominent in autotrophs. This suggests that ethanol supplementation permits *Euglena* to channel excess acetyl-CoA from ethanol catabolism into lipid elongation and desaturation pathways, even as photosynthetic carbon input continues in parallel. Heterotrophic cultures (ethanol as sole carbon, no light) underwent the most dramatic metabolic reorganization compared to autotrophic controls. Deprived of light, *E. gracilis* largely shut down chloroplast-associated metabolism (e.g., we detected minimal levels of chlorophyll derivatives in heterotrophs) and instead activated pathways to efficiently assimilate and store the 2-carbon units from ethanol [25]. Ethanol metabolism in *Euglena* is expected to proceed via alcohol dehydrogenase and acetaldehyde dehydrogenase to produce acetyl-CoA, feeding into the TCA cycle or the glyoxylate cycle for biosynthesis [9,32]. Consistent with this, our data showed heterotrophic enrichment of TCA intermediates and glyoxylate cycle-related acids, supporting the operation of acetate assimilation routes in the dark. A striking outcome of ethanol utilization was the accumulation of storage carbohydrates: heterotrophic cells accumulated significantly higher levels of paramylon (β-1,3-glucan) than either autotrophs or mixotrophs, as evidenced by both our metabolomic signals (e.g., elevated glucose polymers or diagnostic fragments) and prior studies that directly measured paramylon content [20]. In fact, Gulk et al. observed that ethanol supplementation strongly stimulates paramylon accumulation in *E. gracilis* [28].

Another fate of excess acetyl-CoA in the dark was lipid biosynthesis, particularly of neutral storage lipids. Ethanol-grown heterotrophic *Euglena* showed a pronounced increase in lipid-derived metabolites and fatty acyl moieties compared to autotrophic cells. Some of these were identified as wax ester constituents, suggesting that *Euglena* may convert ethanol into wax esters under our culture conditions. Indeed, ethanol has been reported to promote wax ester biosynthesis in *E. gracilis*, alongside paramylon accumulation. Wax ester production is a known fermentative strategy of *Euglena* to store energy under anaerobic or carbon-excess conditions [7]. Although cultures were maintained under aerobic conditions, the combination of darkness and ethanol likely activated storage-related lipid metabolism. The heterotrophic metabolome reflected signs of redox adjustment and stress adaptation, likely driven by ethanol catabolism. Elevated levels of amino acids such as proline suggest roles as osmoprotectants or redox buffers. Notably, putrescine—a stress-responsive polyamine—was more abundant under heterotrophy, potentially serving regulatory or protective functions in response to dark, ethanol-rich conditions. Overall, the heterotrophic state represents a metabolic reprogramming in *E. gracilis*, characterized by the suppression of light-dependent pathways and the activation of carbon storage, redox regulation, and stress mitigation mechanisms.

In summary, the presence or absence of light and ethanol dynamically reshapes the metabolic architecture of *E. gracilis*. Autotrophy maintains pigment and antioxidant biosynthesis through light-driven metabolism. Heterotrophy reprograms cellular metabolism toward storage compound accumulation and redox balancing. Mixotrophy produces a rich biochemical repertoire by integrating light and carbon availability, favoring the biosynthesis of both photo-derived and heterotrophic metabolites. Our findings offer a metabolite-level perspective of this plasticity, underscoring the adaptability of *E. gracilis* and providing valuable insights for biotechnological applications targeting pigment, lipid, and antioxidant production under controlled trophic regimes.

### 3.2. Metabolomic Profiling Reveals That E. gracilis Modulates Its Metabolism Drastically Across Trophic Modes, Yielding Distinct Classes of Enriched Metabolites with Potential in Biotechnological Implications

Under photoautotrophic growth, the cells accumulate a broad array of antioxidant and membrane-stabilizing compounds—notably pigments, flavonoid phenolics, and glycosylated lipids—which serve to quench reactive oxygen species and reinforce chloroplast membranes [33,34]. In contrast, heterotrophic cultivation (with ethanol as carbon source) produces a comparatively narrower metabolite spectrum geared toward essential maintenance: metabolites associated with nitrogen assimilation (e.g., amino acids) and redox balancing are prominent, reflecting efficient incorporation of available nitrogen and adjustments to maintain NAD⁺/NADH equilibrium during ethanol catabolism [35]. This regime also induces pathways for ethanol-linked detoxification as *Euglena* actively metabolizes and neutralizes acetaldehyde—the toxic intermediate of ethanol—via robust alcohol/aldehyde dehydrogenase activity [36].

Mixotrophic cells exhibited the most chemically diverse metabolite profile, reflecting a hybrid metabolic state. These cells retained photosynthesis-derived antioxidants and glycolipids while also synthesizing heterotrophic-associated compounds. Notably, mixotrophic growth uniquely supported the accumulation of complex bioactive metabolites, such as saccharolipids and terpene glycosides, which were not abundant under strictly autotrophic or heterotrophic conditions. This highlights mixotrophy as a promising strategy for enhancing both biomass and biochemical diversity. Nevertheless, autotrophic conditions yielded the highest levels of antioxidant-rich compounds (e.g., carotenoids and tocopherols) [17], whereas heterotrophic growth provides a more focused biochemical state, favoring nitrogen-containing compounds. Together, these findings highlight the metabolic plasticity of *E. gracilis* and guide the choice of cultivation mode for targeted production of valuable compounds in biotechnology.

A key finding of this study is the differential accumulation of bioactive compound classes—especially phenolics, flavonoids, lipids, and terpenoids—across the three trophic modes. These shifts reflect both physiological adaptation and potential for targeted metabolite production. Phenolic and flavonoid compounds were markedly more abundant under photoautotrophic and mixotrophic conditions, particularly under light exposure. Notable examples include cinnamic acid and benzoic acid derivatives, as well as flavone glycosides. These light-dependent patterns likely represent photoprotective responses, as phenolic antioxidants mitigate ROS generated during photosynthesis. Similar trends have been reported in other microalgae, such as *Micractinium* sp., where mixotrophic conditions promoted higher total phenolic content (e.g., gallic acid reaching ~469 µg/g) compared to heterotrophy [37].

In our dataset, heterotrophic cultures produced fewer phenolic compounds, though some (e.g., hydroxybenzoic acids) accumulated at higher levels, suggesting selective retention of basic ROS-scavenging metabolites in the absence of light. These observations support the hypothesis that chloroplast-dependent or light-responsive nuclear pathways in *E. gracilis* activate the shikimate pathway, generating aromatic amino acid precursors for secondary metabolite biosynthesis [9]. The presence of flavonoids under autotrophic and mixotrophic growth may also reflect the organism’s evolutionary heritage from green algal plastids or stress-induced signaling pathways. Collectively, our results confirm that light exposure—alone or in combination with external carbon—enhances the phenolic and flavonoid profile in *E. gracilis*, thereby offering leverage for maximizing antioxidant production in algal bioprocesses.

Terpenoid metabolites, including carotenoid pigments and isoprenoid vitamins, also displayed trophic-dependent differences. *E. gracilis* is known to synthesize several carotenoids (β-carotene, neoxanthin, diadinoxanthin, lutein, etc.) as part of its photosynthetic apparatus, as well as antioxidant terpenoids like α-tocopherol (vitamin E) in chloroplast membranes [38]. In our metabolomic analysis, β-carotene itself was not confidently detected, but several xanthophylls and their derivatives, such as diadinoxanthin and heteroxanthin-related compounds, were observed, especially under photoautotrophic conditions. These findings suggest that xanthophyll-based photoprotective mechanisms remain active in the light-dependent metabolic states. As expected, chloroplast-associated pigment biosynthesis was downregulated under heterotrophic conditions, leading to reduced or undetectable carotenoid and chlorophyll signals, consistent with the bleached phenotypes observed in long-term dark-grown cultures [39].

Mixotrophic cultures displayed an intermediate pigment profile, retaining light-dependent pigments but at lower levels than strictly autotrophic cells. This may reflect reduced photosystem activity due to partial reliance on ethanol, as previously observed in mixotrophic *Euglena* with diminished pigment levels by day 12 [17]. Interestingly, ethanol supplementation did not significantly upregulate carotenoid production beyond autotrophic levels, suggesting that it is not a strong stressor or inducer of pigment biosynthesis under light [28]. However, subtle shifts in pigment composition, such as increased photoprotective xanthophylls, may serve to counteract oxidative stress associated with simultaneous light and ethanol metabolism.

In summary, autotrophic conditions support robust biosynthesis of terpenoid pigments and isoprenoid antioxidants due to active chloroplast function, whereas heterotrophy suppresses these pathways, potentially redirecting precursors to other isoprenoid branches like sterol synthesis. Mixotrophy preserves partial pigment production and combines light- and ethanol-responsive metabolic features. The distinct terpenoid profiles observed across trophic modes demonstrate *E. gracilis*’ capacity to re-tune secondary metabolism in response to environmental inputs. From an application standpoint, culture conditions can be strategically modulated to favor pigment production (e.g., for natural antioxidants or colorants) or other high-value secondary metabolites, depending on desired outcomes.

## 4. Materials and Methods

### 4.1. Culture and Biomass Production

*E. gracilis* SAG 1224-5/25 was obtained from the Culture Collection of Algae at the University of Göttingen, Germany (SAG) and maintained in modified Hutner’s medium. Cultures were grown under three trophic conditions: photoautotrophic (light intensity of 300 µmol photons/m^2^/s), heterotrophic (1% *v*/*v* ethanol in complete darkness with flasks wrapped in aluminium foil), and mixotrophic (1% *v*/*v* ethanol with light at 300 µmol photons/m^2^/s). All cultures were initiated at pH 6.5 with a starting cell density of 10,000 cells/mL, maintained at 120 rpm, and cultivated in triplicate. For biomass production, 800 mL cultures were grown in 1 L Erlenmeyer flasks under the same respective conditions. On days 0 and 10, 10 mL of culture was sampled, filtered through pre-weighed fiberglass filters, and dried at 60 °C until constant weight was achieved. Biomass from larger volumes was harvested by centrifugation at 5000× *g* and subsequently lyophilized for downstream analysis.

### 4.2. Total Phenolic and Total Flavonoids Content Analysis

One gram of dried *E. gracilis* biomass from each condition was extracted with 100% methanol by shaking at 900 rpm for 1 h at 25 °C. The extract was then centrifuged at 10,000× *g* for 1 min, and the supernatant was used for analysis. For TPC determination, 25 µL of the extract or gallic acid standard (0.01 mg/mL) was added to a 96-well plate, followed by 125 µL DI water, 25 µL of 95% ethanol, and 12.5 µL of 50% Folin–Ciocalteu reagent. After 5 min, 25 µL of 5% sodium carbonate was added. The plate was incubated in the dark for 1 h, and absorbance was measured at 725 nm using a microplate reader [40]. Total phenolic content was calculated from a gallic acid standard curve. The same extract used for TPC analysis was also used for flavonoid determination. In a 96-well plate, 20 µL of extract or quercetin standard (0.01 mg/mL) was added, followed by 4 µL of 10% aluminum chloride, 60 µL methanol, 4 µL of 1 M potassium acetate, and 112 µL distilled water. The mixture was incubated at room temperature for 30 min. Absorbance was measured at 415 nm. Total flavonoid content was calculated using a quercetin standard curve [41]. The analyses were conducted in biological triplicate, and statistical analyses were performed in R (v4.1.3) using one-way ANOVA (car package) followed by Tukey’s HSD post hoc test (multcomp package), with *p* < 0.05 considered statistically significant.

### 4.3. Metabolomic Sample Preparation

*E. gracilis* biomass samples (10 mg) were dissolved in 600 µL of 70% ethanol containing 25 ng/mL sulfadimethoxine as an internal standard. The samples were vortexed thoroughly to ensure proper dissolution. To monitor analytical performance, a pooled quality control (QC) sample was prepared by aliquoting 60 µL from each sample. The QC sample was further diluted into seven concentration levels: 0%, 1%, 10%, 20%, 50%, 80%, and 100% to evaluate signal linearity and response variability across the analytical workflow. All samples were centrifuged at 14,000 rpm for 10 min, and the resulting supernatant was carefully transferred into LC-MS vials for metabolomic analysis.

### 4.4. Liquid Chromatography-Mass Spectrometry (LC-MS) Analysis

Metabolite separation was performed using an Agilent 1290 Infinity II LC system (Agilent Technologies, Santa Clara, CA, USA) equipped with a Poroshell 120 EC-C18 column (2.1 × 100 mm, 2.7 µm) maintained at 50 °C. The injection volume was set to 10 µL, with a constant flow rate of 0.4 mL/min. A binary solvent system was employed, consisting of mobile phase A (0.1% formic acid in water) and mobile phase B (0.1% formic acid in acetonitrile). A gradient elution program was applied to ensure efficient metabolite separation, starting with 100% A at 0.0 min, followed by a gradual increase of B to 55% at 10.5 min, 75% at 12.5 min, and reaching 100% B at 14.0 min, which was maintained until 17.0 min before re-equilibration to initial conditions by 20.0 min. Mass spectrometry analysis was conducted using an Agilent LC-QTOF 6545XT in both positive and negative ionization modes with high-resolution detection. The electrospray ionization (ESI) conditions were optimized for sensitivity, with a drying gas temperature of 325 °C and a drying gas flow rate of 13 L/min. The sheath gas temperature was set at 275 °C, with a sheath gas flow rate of 12 L/min, while the nebulizer pressure was maintained at 45 psi. Capillary voltage settings were optimized at 4000 V for positive mode and 3000 V for negative mode. The instrument was operated in high-resolution mode, with an MS1 scan range of 40–1700 *m*/*z* and an MS2 scan range of 25–1000 *m*/*z*. Collision-induced dissociation (CID) energy was set at 20 eV for positive mode and 10 eV for negative mode, with an acquisition rate of 3.35 spectra per second to ensure high spectral coverage.

### 4.5. Data Processing and Metabolite Identification

All raw LC-MS data were processed using MS-Dial version 5.3, where spectral alignment was performed using one of the QC samples as a reference. Normalization was carried out using sulfadimethoxine as an internal standard, combined with the LOWESS method to correct for systematic drift. To minimize background noise, predefined mass exclusion criteria were applied, removing common contaminants at 121.0509 *m*/*z* and 922.0098 *m*/*z* in positive mode and at 112.9856 *m*/*z*, 119.0363 *m*/*z*, 966.0007 *m*/*z*, and 1033.9881 *m*/*z* in negative mode. Only [M + H] adducts in positive mode and [M−] adducts in negative mode were considered for further analysis. For metabolite identification, spectral matching was performed against multiple reference libraries, including the MS-Dial ESI(+/−) MS/MS database from authentic standards, the Fiehn/Vaniya Natural Product Library, and the BMDMS-NP (Bio-Molecular Discovery MS Natural Products Database). Metabolites with an identification score of ≥0.70 were retained for downstream analysis. To ensure robust data quality, pre-filtration criteria were applied, requiring a Pearson correlation coefficient of ≥0.70 between features and dilution QC samples. Additionally, only metabolites with a coefficient of variation (CV) of <30% in QC samples were included. This filtering process resulted in 3561 features in positive mode and 2388 features in negative mode, forming the final dataset for statistical analysis. A detailed list of identified metabolites is provided in the Supplementary Data file.

### 4.6. Statistical Analysis

Data analysis was conducted using MetaboAnalyst 6.0 [42]. To account for missing values, zero values were imputed with 1/5th of the minimum positive value of each variable. The dataset was then log10-transformed to normalize intensity distributions prior to multivariate statistical analysis. Principal Component Analysis (PCA) was applied to explore global metabolic variance across samples, while Sparse Partial Least Squares Discriminant Analysis (sPLS-DA) was performed to identify discriminant metabolic features contributing to trophic-specific metabolic profiles. This untargeted metabolomics workflow integrates high-resolution LC-QTOF-MS, advanced spectral library matching, and multivariate statistical approaches to systematically explore the metabolic adaptations of *E. gracilis* under different trophic conditions. By combining multiple ionization modes, authentic standard databases, and stringent QC-based filtering, the methodology ensures high-confidence metabolite annotation and reproducibility, providing a reliable platform for elucidating metabolic shifts associated with autotrophic, mixotrophic, and heterotrophic metabolism.

### 4.7. Comparative Metabolomics and Differential Metabolite Analysis

Heatmap analysis was performed based on hierarchical clustering to visualize relative metabolite abundance across autotrophic (P), mixotrophic (M), and heterotrophic (H) conditions, allowing identification of condition-specific patterns. To further assess overall metabolic similarity between conditions, neighbor-joining (NJ) clustering was conducted using Euclidean distance on the combined positive and negative mode datasets. For comparative analysis, metabolite data were first filtered to ensure reliability and biological relevance using the following criteria: signal-to-blank ratio ≥ 10, coefficient of variation (CV%) ≤ 30%, and signal intensity ≥ 10,000. Three-way Venn diagrams were constructed to identify shared and unique metabolites across trophic conditions. Unique features were manually annotated based on metabolite names and ontologies and assigned to metabolic pathways. Differentially expressed metabolites (DEMs) were identified using volcano plot analysis, applying a cutoff of *p* ≤ 0.05 and |log_2_FC| ≥1. From each pairwise comparison (P vs. M, P vs. H, and M vs. H), the top 20 DEMs with the highest absolute log_2_FC values were selected for further analysis and visualization.

## 5. Conclusions

This study demonstrates that trophic mode plays a critical role in shaping the metabolic architecture of *E. gracilis*. Untargeted metabolomic profiling revealed distinct metabolic signatures under photoautotrophic, mixotrophic, and heterotrophic conditions. Photoautotrophic growth favored the accumulation of antioxidant pigments, flavonoids, and membrane-stabilizing lipids, supporting photosynthetic function and oxidative stress protection. Heterotrophic growth, driven by ethanol supplementation, induced a shift toward storage compound accumulation, nitrogen assimilation, and redox balancing pathways. Mixotrophic cultures exhibited a blended but distinct metabolic profile, integrating both light-driven and ethanol-derived metabolic activities and promoting enhanced diversity of secondary metabolites. Differential metabolite analysis and pathway enrichment further confirmed that each trophic mode activates specific biosynthetic strategies suited to its energy and environmental demands. The ability of *E. gracilis* to flexibly reprogram its metabolism highlights its potential as a sustainable platform for producing bioactive compounds, functional foods, and renewable biomaterials. These findings provide valuable insights for optimizing cultivation strategies and developing biotechnological applications that align with future goals in food security, health promotion, and clean energy solutions.

## Data Availability

Data are contained within the article and Supplementary Files.

## Supplementary Materials

The following supporting information can be downloaded at: https://www.mdpi.com/article/10.3390/plants14111580/s1, Supplementary Data S1: Data S1; Supplementary Data S2: Data S2.
